# PKN3 is the major regulator of angiogenesis and tumor metastasis in mice

**DOI:** 10.1038/srep18979

**Published:** 2016-01-08

**Authors:** Hideyuki Mukai, Aiko Muramatsu, Rana Mashud, Koji Kubouchi, Sho Tsujimoto, Tsunaki Hongu, Yasunori Kanaho, Masanobu Tsubaki, Shozo Nishida, Go Shioi, Sally Danno, Mona Mehruba, Ryosuke Satoh, Reiko Sugiura

**Affiliations:** 1 Biosignal Research Center, Kobe University, Kobe 657-8501, Japan; 2 Graduate School of Science and Technology, Kobe University, Kobe 657-8501, Japan; 3 Graduate School of Medicine, Kobe University, Kobe 657-8501, Japan; 4 Laboratory of Molecular Pharmacogenomics, School of Pharmaceutical Sciences, Kinki University, 3-4-1 Kowakae, Higashi-Osaka 577-8502, Japan; 5 Graduate School of Comprehensive Human Sciences, Institute of Basic Medical Sciences, University of Tsukuba, Ibaraki 305-8575, Japan; 6 Division of Pharmacotherapy, Kinki University School of Pharmacy, Kowakae, Higashi-Osaka 577-8502, Japan; 7 Genetic Engineering Team, Division of Bio-function Dynamics Imaging, RIKEN Center for Life Science Technologies (CLST), 2-2-3 Minatojima Minami,Chuou-ku, Kobe 650-0047.

## Abstract

PKN, a conserved family member related to PKC, was the first protein kinase identified as a target of the small GTPase Rho. PKN is involved in various functions including cytoskeletal arrangement and cell adhesion. Furthermore, the enrichment of PKN3 mRNA in some cancer cell lines as well as its requirement in malignant prostate cell growth suggested its involvement in oncogenesis. Despite intensive research efforts, physiological as well as pathological roles of PKN3 *in vivo* remain elusive. Here, we generated mice with a targeted deletion of PKN3. The PKN3 knockout (KO) mice are viable and develop normally. However, the absence of PKN3 had an impact on angiogenesis as evidenced by marked suppressions of micro-vessel sprouting in *ex vivo* aortic ring assay and *in vivo* corneal pocket assay. Furthermore, the PKN3 KO mice exhibited an impaired lung metastasis of melanoma cells when administered from the tail vein. Importantly, PKN3 knock-down by small interfering RNA (siRNA) induced a glycosylation defect of cell-surface glycoproteins, including ICAM-1, integrin β1 and integrin α5 in HUVECs. Our data provide the first *in vivo* genetic demonstration that PKN3 plays critical roles in angiogenesis and tumor metastasis, and that defective maturation of cell surface glycoproteins might underlie these phenotypes.

Protein kinase N (PKN) is a serine/threonine protein kinase with a catalytic domain homologous to protein kinase C and a unique regulatory region containing antiparallel coiled-coil (ACC) domains^1^,^2^. PKN is composed of three isoforms (PKN1, PKN2, and PKN3) derived from different genes in mammals. PKN1 and PKN2 are widely distributed in the mammalian tissues^1^. In contrast, previous reports showed that PKN3 mRNA was almost undetectable in normal adult tissues, but was found upregulated in various cancer cell lines^3^. PKN was first described as a fatty acid- and phospholipid- activated serine/threonine protein kinase and also as a protease- activated protein kinase^4^,^5^, however, the responsiveness of protein kinase activity to phospholipids and fatty acids *in vitro* differ in each isoform^1^, and PKN2 and PKN3 are much less responsive to arachidonic acid than PKN1^3^,^6^. PKN was also the first identified effector protein kinase of Rho GTPase, and each PKN isoform has been reported so far to bind to various Rho family GTPases in mammalian tissues^1^,^3^,^7^,^8^,^9^,^10^,^11^,^12^,^13^,^14^. Members of the Rho family of small GTPases are known to serve as molecular switches that regulate a diverse set of cellular functions including cell migration, polarization, adhesion, cell-cycle control, apoptosis, cellular transformation and metastasis^15^,^16^,^17^. PKN isoforms have been postulated to play some roles in the functions of these Rho family GTPases.

So far, there have been accumulated reports about the potential function of PKN isoforms using cultured cell experiments such as; involvement in the regulation of cytoskeletal reorganization^12^,^18^, cell adhesion^19^,^20^, cell-cycle regulation^21^,^22^,^23^, and tumorigenesis^24^,^25^. However, only a few organismal level studies elucidating the physiological function of PKN *in vivo* have been conducted. As an example, the Drosophila Pkn protein, single PKN ortholog encoded by the Drosophila genome, is required specifically for the migration and adhesion of the epidermal cells during the morphogenetic process of dorsal closure of the embryo, a developmental process in which Rho and Rac GTPases have been directly implicated^26^. Since mammalian PKN isoforms, thus having overlapping expression profile and catalytic activity, it is essential for the clarification of the physiological function of each isoform to specifically abrogate each signaling pathway in animal level *in vivo*. With regard to the organismal study for each mammalian PKN isoform, only one KO mouse line of PKN1 has been generated, and only abnormal survival and selection of B-cells in secondary lymphoid organs have been reported so far in these mice^27^, without referring to the relationship of PKN1 with cell migration or with Rho GTPases. Here we focus on another isoform, PKN3, and describe the generation and characterization of the KO mouse line of this isoform. Fibroblastic cells derived from PKN3 KO mice have lower cell migration activity, and PKN3 KO mice showed poor growth factor-induced angiogenesis and suppression of lung metastasis of B16 melanoma cells injected through tail vein. The relationship between the suppression of growth factor-induced angiogenesis and tumor metastasis has not been clarified, but the PKN3 KO mouse experiment clearly revealed that the host PKN3 is crucial for tumor metastasis. Therefore, targeting PKN3 in host stromal cells has a therapeutic potential for the prevention of cancer metastasis. We also discuss the potential relationship between PKN3 and the small GTPase RhoC based on the phenotype induced by deficiency of each molecule.

## Results

### PKN3 distribution in normal adult mouse tissues

**Figure 1:**
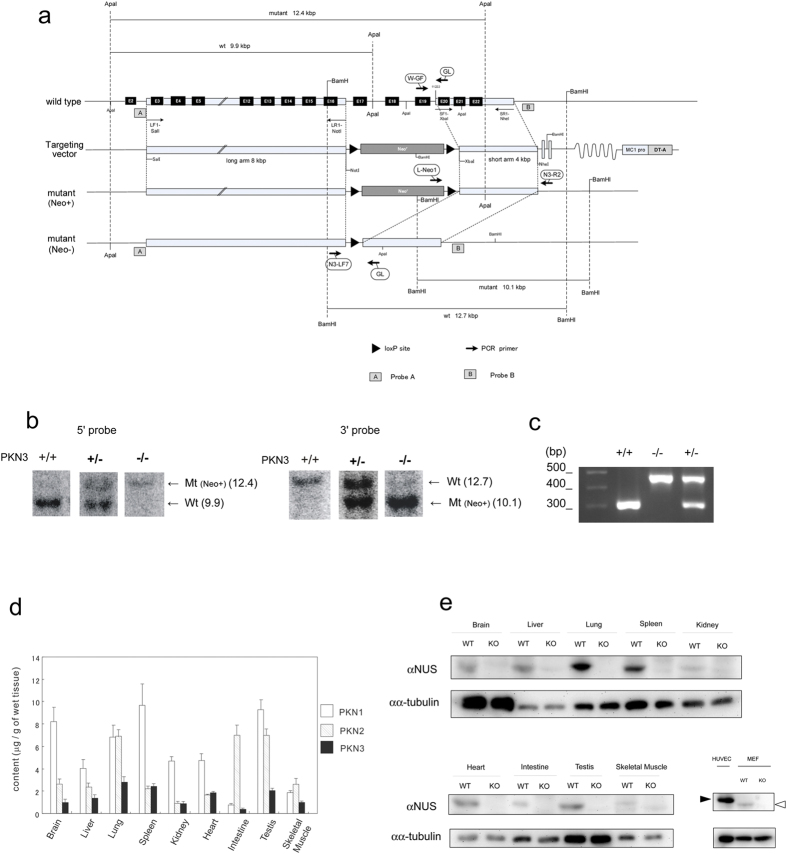
Generation of PKN3 KO mice. (**a**) Scheme of PKN3 genomic DNA, targeting vector, and disrupted gene. The targeting vector and a partial map of the PKN3 locus before and after homologous recombination in ES cells, and after further deletion of neomycin resistance cassette by Cre mediated recombination. Positions of the loxP sites are designated by black triangles. Subsequent breeding of heterozygous mice indicated by PKN3 +/− generates PKN3 knockout (KO) mice (PKN3 −/−). The exons, deduced by comparison with the cDNA sequence, are denoted by black boxes. The positions of the genomic DNA probes (**a**,**b**) used in Southern blotting are indicated, as well as the positions of the primers used for screening of homologous recombination (L-Neo1 and N3-R2), and subsequent PCR genotyping (W-GF, GL, N3-LF7). (**b**) Southern blot analysis. Shown is the result of a representative litter of F2 mice obtained by crossing a pair of PKN3 +/− (containing Neo cassette) F1 mice. Genomic DNA was digested with *Apa*I and probed with probe A on the left, and digested with *Bam*HI and probed with probe B on the right. (**c**) PCR genotyping for discrimination between WT and mutant allele lacking Neo cassette. (**d**) Quantification of PKN3 in various tissues of WT mice. Each tissue was homogenized and subjected to SDS-PAGE. The amount of PKN3 was measured by immunoblot analysis using the αNUS antibody and indicated as “μg/g of wet tissue”. The data are expressed as the means ± S.E.M from n = 3 mice. (**e**) Expression of PKN3. Whole-cell lysates of 500 μg wet weight of each tissue from WT and PKN3 KO mice was resolved by SDS-PAGE, and subjected to immunoblot analysis using the αNUS antibody and anti α-tubulin antibody. Fifty μg protein of whole cell lysate of each HUVECs and mouse embryonic fibroblasts was subjected to SDS-PAGE followed by immunoblot analysis using the αNUS antibody and anti α-tubulin antibody. White arrowhead indicates mouse PKN3. Black arrowhead indicates human PKN3. WT, WT mice; KO, PKN3 −/− mice.

PKN1 has been reported to be expressed in almost every tissue and is particularly enriched in the spleen, thymus, testes, and brain (PKN1 is approximately 0.01% of total protein in the rat brain)^28^. On the contrary, initial experiment by Northern blotting reported that PKN3 mRNA was almost undetectable in normal tissues compared to cancer cell lines^3^. In order to increase the sensitivity and also to permit analysis of PKN3 mRNA expression, real-time quantitative reverse-transcriptase PCR was employed with oligonucleotides specific for mouse PKN3 (Supplementary Fig. 1). This analysis was performed on a variety of adult mouse tissues and revealed ubiquitous expression, indicating that PKN3 mRNA is not limited to tumor cells. Since the expression level of PKN3 protein as well as PKN2 protein in various normal tissues has not been reported yet, next we measured the protein amount of all the isoforms of PKN by the immunochemical method as shown in Fig. 1d. PKN3 protein is also ubiquitously expressed in normal adult tissues whereas the amount of PKN3 is less than that of PKN1 and PKN2 in most of the tissues.

### PKN3 KO mice are viable without any obvious phenotype

In order to identify the physiological functions of PKN3 expressed in normal tissues, we established a mutant mouse strain deficient in PKN3. The targeting vector was designed to replace the exons 17–19 (encoding the catalytic region containing the activation loop) by a neomycin-phosphoglycerate kinase (NEO-PGK) cassette (Fig. 1a). TT2 embryonic stem cell clones generated by homologous recombination were injected into ICR 8-cell stage embryos to generate chimeric males. Heterozygous mice from the F1 generation were intercrossed to generate PKN3 KO mice that were obtained in the Mendelian ratio. We checked mice genotypes by Southern blotting and PCR (Fig. 1b,c). The expression of PKN3 was determined by immunoblotting (Fig. 1e and Supplementary Fig. 2). As expected, tissues from PKN3 KO mice did not produce any PKN3 fragment, as assessed by immunoblotting using antibodies against PKN3. Expression of PKN1 and PKN2, the other isoforms of the PKN family, was not significantly changed (data not shown). Despite general PKN3 deficiency, PKN3 KO mice developed to fertile adults indistinguishable from those of their wild type (WT) counterparts. These findings suggest that PKN3 is dispensable for development and growth to the adult stage.

### Functional Characterization of embryonic fibroblast from PKN3 KO mice

**Figure 2:**
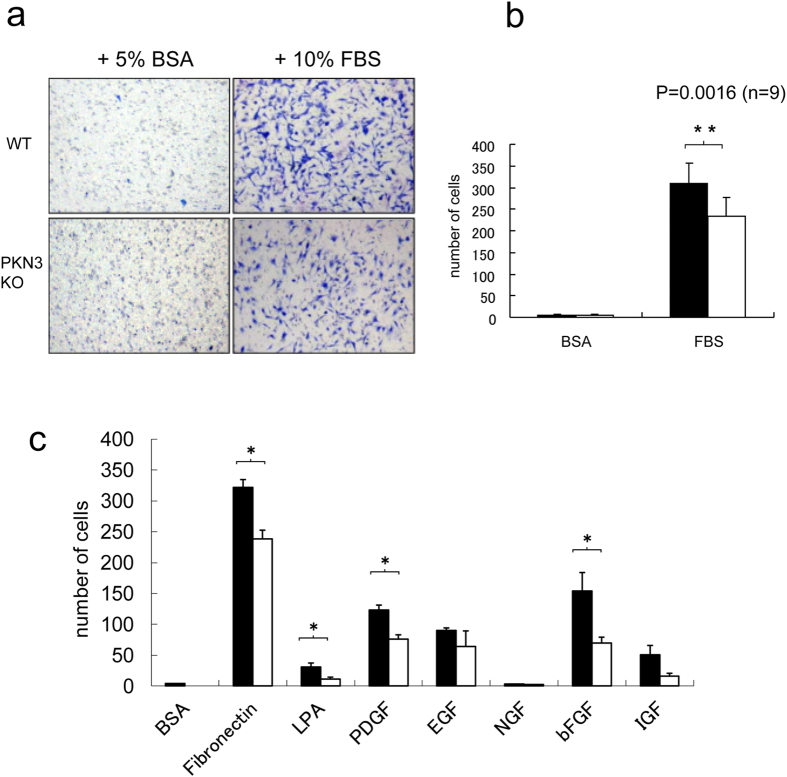
Migration of embryonic fibroblasts from WT and PKN3 KO mice. (**a**) Cell migration was determined using the Transwell membrane. After 5 h of incubation at 37 °C, the cells that had migrated to the lower surface of the membrane were fixed and stained. Shown are the representative photos of lower surface of the Transwell membrane. (**b**) Diagrams of migrating cells in the presence of 10% FBS or 5% BSA as a control. n = 9. ** indicates P < 0.01. (**c**) Diagrams of migrating cells in the presence of various migration factors. The concentration of migration factors are as follows. Fibronectin, 10 μg/ml; lysophosphatidic acid (LPA), 10 μM; PDGF, 25 ng/ml; Epidermal growth factor (EGF), 25 ng/ml; bFGF, 20 ng/ml; Nerve growth factor (NGF), 50 ng/ml; Insulin-like growth factor-1 (IGF-1), 100 ng/ml. P values are as follows. Fibronectin, p = 0.044/n = 9; LPA, p = 0.029/n = 4; PDGF, p = 0.035/n = 9; bFGF, p = 0.036/n = 4; IGF-1, p = 0.069/n = 4. * indicates P < 0.05.

PKN3 was reported to bind to, RhoA, RhoB, and RhoC, the Rho family small GTPases^3^,^13^,^14^, and also bind to GRAF (GTPase-activating protein for Rho associated with focal adhesion kinase) and GRAF2^29^ which are suggested to be involved in cytoskeletal rearrangement of cells. Actually, PKN3 has been suggested to be involved in cell migration in PC-3 human prostate cancer cell line^25^ and human umbilical vascular endothelial cell (HUVEC)^30^, by experiments using small hairpin RNA (shRNA) or small interfering RNA (siRNA) against PKN3, respectively. In order to confirm and further explore the role of PKN3 in regulation of cell motility, embryonic fibroblast cells were isolated from WT and PKN3 KO mouse embryos. The embryonic fibroblasts from WT mice express PKN3 as shown in Fig. 1e. PKN3 KO cells exhibited lower migratory activity under transwell migration assay using a Boyden chamber as compared to WT cells (Fig. 2). Inhibition was observed in cell migration induced by various growth factors, suggesting that PKN3 is involved in relatively direct regulation of the actin cytoskeleton of primary fibroblastic cells (Fig. 2c).

### PKN3 deletion impairs angiogenesis

**Figure 3:**
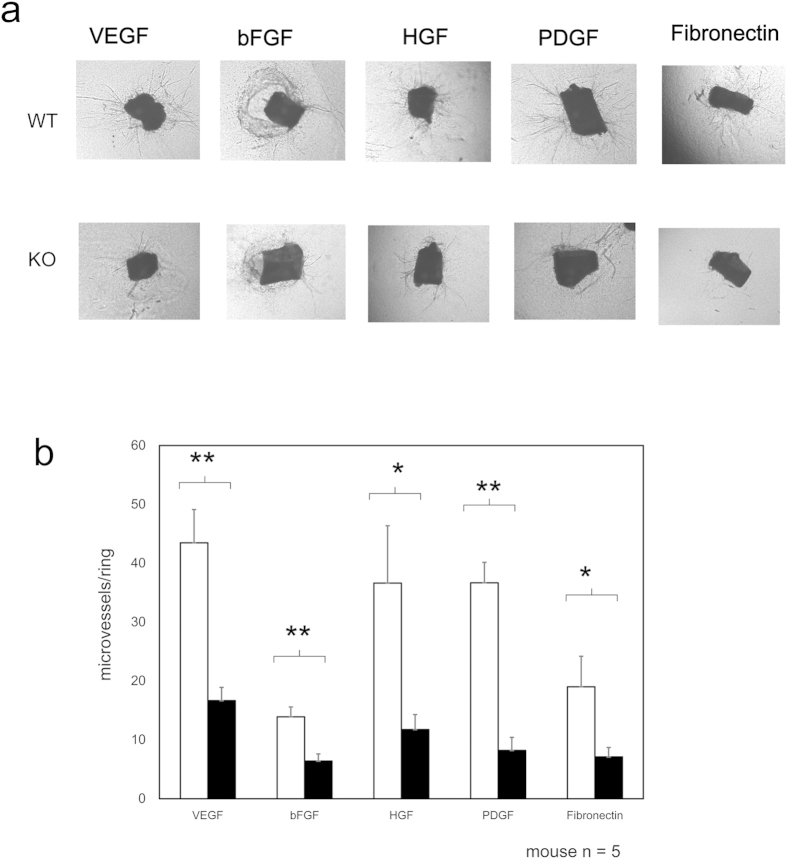
Influence of PKN3 KO in the regulation of *ex vivo* angiogenesis. (**a**) Abdominal aortic ring segments from WT or PKN3 KO mice embedded in matrigel (for PDGF) or collagen (for VEGF, bFGF, HGF, and Fibronectin). Aortic ring segments were incubated with each growth factor indicated for 6 days. Panel shows representative photomicrographs of microvascular sprouting in each condition after 6 days in culture. (**b**) Effect on the sprouting vessels from *ex vivo* aortic rings. Bars represent mean of 15 independent experiments ± SEM. (mouse number of each genotype is 5). * and ** indicate P < 0.05 and P < 0.01, respectively.

Since the cell migration of fibroblastic cells and vascular endothelial cells are known to be important in angiogenesis^31^, and HUVECs express PKN3 as shown in Fig. 1e, we next examined the importance of PKN3 in growth factor-mediated angiogenesis using this mouse line. Mouse “aortic ring” explants in collagen or matrigel *ex vivo* are known to induce capillary-like structures^32^. Then aortic rings, isolated from WT and PKN3 KO mice, were treated with each growth factor such as vascular endothelial growth factor (VEGF), basic fibroblast growth factor (bFGF), hepatic growth factor (HGF), platelet derived growth factor (PDGF), and fibronectin, and the number of emerging microvessel sprouts was counted after seven days in three-dimensional culture. Aortic rings from PKN3 KO mice showed impaired microvascular sprouting compared with WT controls (representative photographs are shown in Fig. 3a, and statistical analysis is shown in Fig. 3b). These data provide evidence that PKN3 is involved in the regulation of the growth factor-mediated angiogenesis.

**Figure 4:**
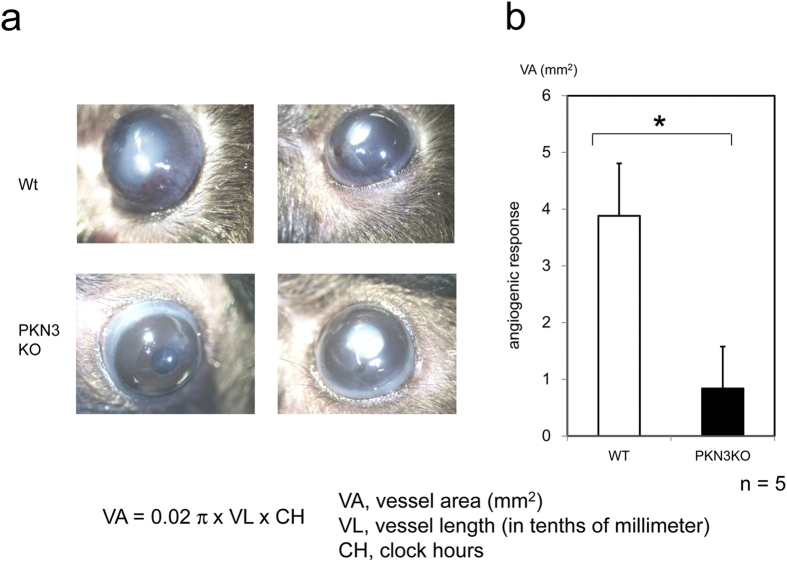
Corneal angiogenesis assay of PKN3 KO mice. (**a**) Corneas of mice 7 days after post implantation of growth factor pellets. A hydron pellet containing bFGF was implanted in a surgically created micropocket on the cornea of PKN3 KO mice and WT control mice. (**b**) Angiogenic response quantified by measuring the neovascular area in the corneas. Five pairs of animals were used in the study.

To gain insight into the role of PKN3 during angiogenesis *in vivo*, we used a corneal micropocket assay^33^. After seven days of exposure to an implanted pellet containing bFGF, corneas of WT mice showed limbic vessel dilation and sprouting (representative photographs are shown in Fig. 4a, and statistical analysis is shown in Fig. 4b). In contrast, corneal vessels from PKN3 KO mice showed tendency of minimal bFGF-stimulated sprouting, as evidenced by reduction in vessel area (Fig. 4). Despite the deficiency of *in vivo* angiogenesis in the corneal pocket assay, PKN3 KO mice developed to the adult stage without obvious vascular deficiencies and developmental defects as described above. These results indicate that PKN3 is not needed for normal vascular development but supports angiogenesis in some circumstances. This might be due to the successful compensation for the lack of PKN3 in mouse development with PKN1 and PKN2 expressed abundantly in mouse tissues, and the extra demand of angiogenesis might absolutely require PKN3.

### PKN3 KO does not affect tumor angiogenesis

**Figure 5:**
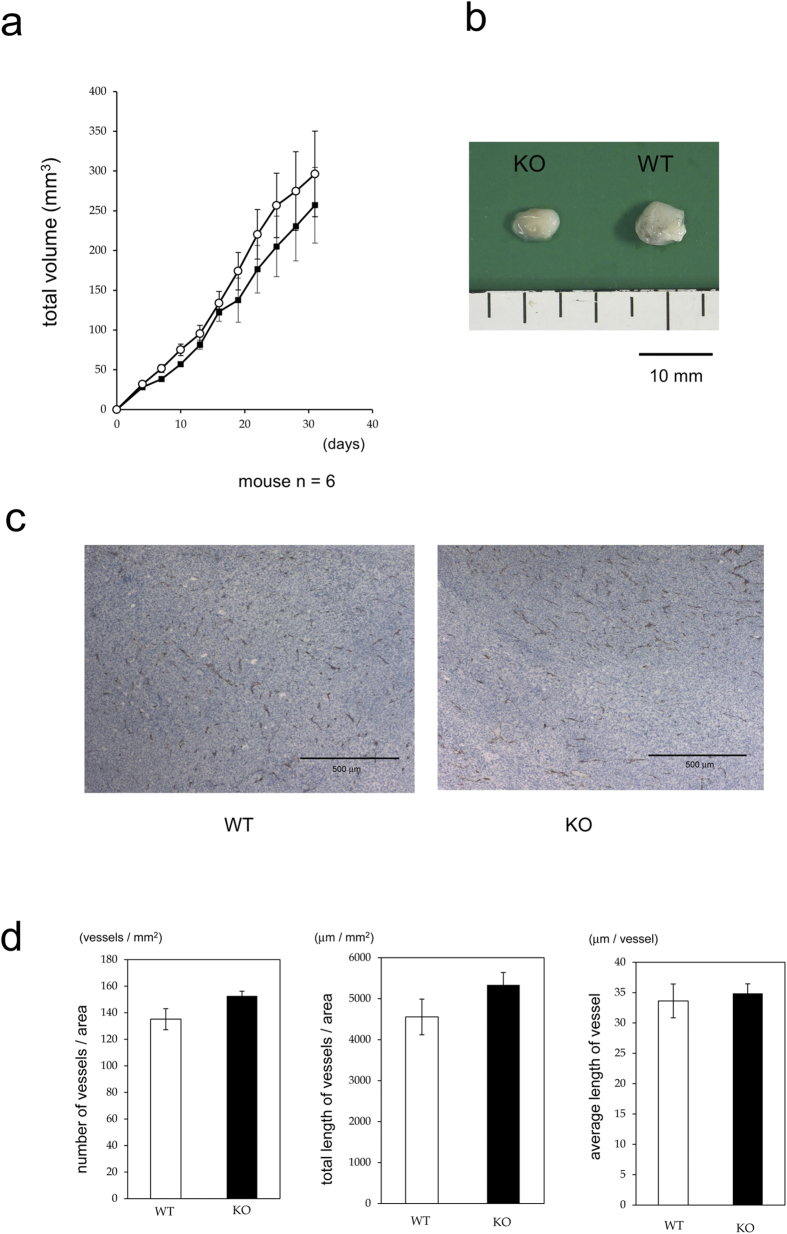
Growth of the primary tumor. (**a**) Growth kinetics of implanted Lewis lung cancer in WT and PKN3 KO mice. The closed square indicates WT and the open circle indicates PKN3 KO mice. n = 6 mice per group. Error bar, SEM. (**b**) Representative photograph of tumors when resected. (**c**) Representative photographs of anti CD31 antibody staining of tumor sections. (**d**) The density and the length of blood vessels in tumors. The entire field of maximum tumor sections were digitalized by CCD camera and the CD31 staining were traced and quantified by using WinROOF (Ver7.2, Mitani corporation, Fukui, Japan) software. Each data point represents the mean ± SE. n = 6 mice per group.

Alterations in aortic ring outgrowth are typically accompanied by altered tumor angiogenesis^34^,^35^,^36^. To determine whether stromal PKN3 expression affects tumor growth and angiogenesis, we injected PKN3 KO and WT mice subcutaneously with 1 × 10^6^ Lewis lung carcinoma cells and monitored tumor growth over time. Calliper measurements of the tumors showed no significant difference in tumor sizes between the WT controls and PKN3 KO mice (Fig. 5a). We next analyzed the blood vessel density of the tumors in WT and PKN3 KO mice. Size-matched, 30-day-old tumors from WT and PKN3 KO mice were taken and the number and the total length of CD31-positive blood vessels per unit area across entire midline sections was assessed. None of the density, total length per unit area, and the average length of each blood vessel were significantly different in PKN3 KO mice compared with WT controls (representative CD31 staining images are shown in Fig. 5c, and statistical analysis is shown in Fig. 5d), suggesting that PKN3 KO is not sufficient to inhibit tumor angiogenesis.

### Melanoma metastasis can be restrained in mice lacking PKN3

**Figure 6:**
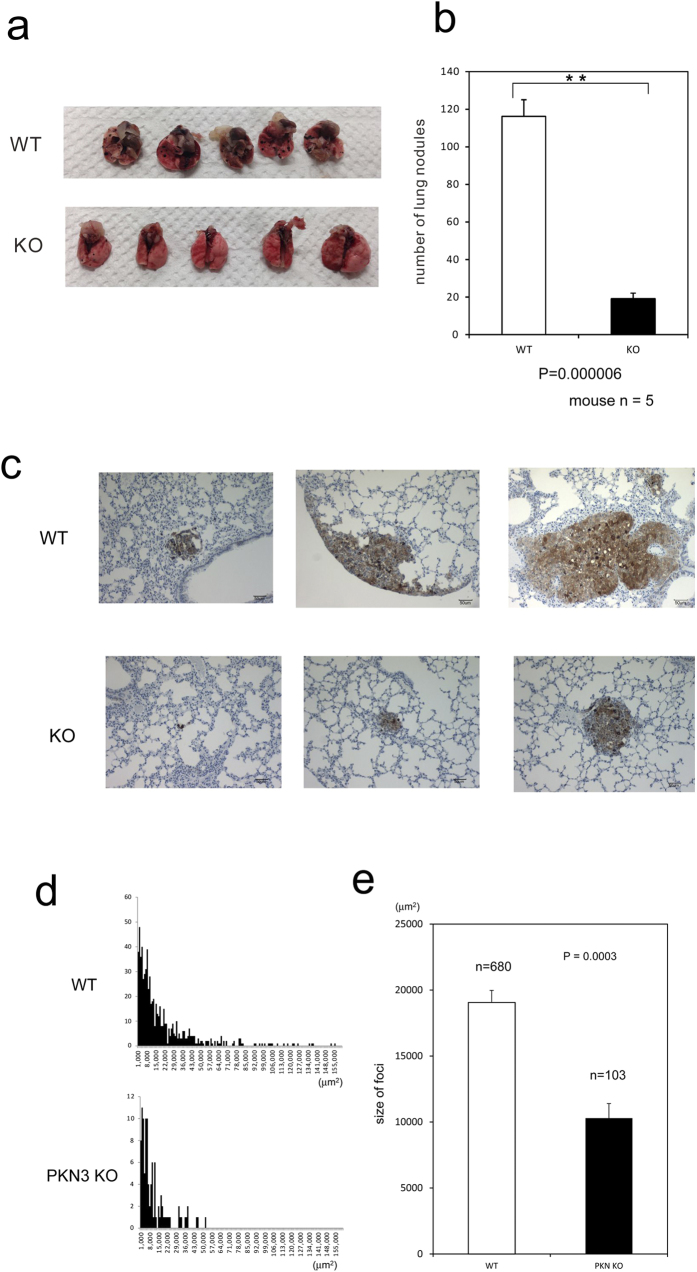
Reduced metastasis formation in PKN3 KO mice. (**a**) Macroscopic appearance of lungs obtained from WT and PKN3 KO mice at 14 days after an i.v. injection of B16BL6 cells. (**b**) The number of metastatic foci on the lung surface. ** indicates P < 0.01. (**c**) Microscopic appearance of lungs after B16BL6 cell injection. Lungs were removed at 14 days after injection of melanoma cells, and processed to anti S100 antibody staining to visualize melanoma cells and H&E staining. Representative results from independent mice are shown here. (**d**) The tumor size histogram of metastatic foci. The horizontal axis indicates the size of tumor (μm^2^) observed in the slice section of mouse lungs. The vertical axis indicate the number of foci. (**e**) The average size of foci of mouse lungs.

Tumor derived PKN3 was previously reported to be involved in metastasis of PC-3 prostate cancer cells by an experiment using PKN3 shRNA treatment of this cell line^25^. However, it is not clear whether PKN3 in the host stromal cells has a role in tumor metastasis. Santel *et al.* have suggested that knockdown of PKN3 by giving the siRNA drug, Atu027, into whole body significantly suppressed tumor metastasis in mouse model^37^, which suggests the potential role of host PKN3 in tumor progression, whereas clear deprivation of the effect of siRNA on tumor-derived PKN3 seems to be impossible in this system. Thus, to clearly assess the role of host PKN3 in tumor metastasis, B16BL6 melanoma cells were injected into the tail vein of WT and PKN3 KO mice. Fourteen days later, mice were sacrificed and lungs were observed from outside. Suppression of metastases was observed in the lungs of PKN3 KO mice as compared to WT mice (Fig. 6a,b). These lungs were dissected and further analyzed microscopically. The lung sections also showed significantly less number of metastatic foci visible in the lung of PKN3 KO mice as compared to WT mice (Fig. 6c). The average size of the metastatic foci was significantly smaller in PKN3 KO mouse lungs than WT lungs (Fig. 6d,e). Taken together, these data demonstrated that stromal PKN3 plays important roles in metastasis of melanoma.

**Figure 7:**
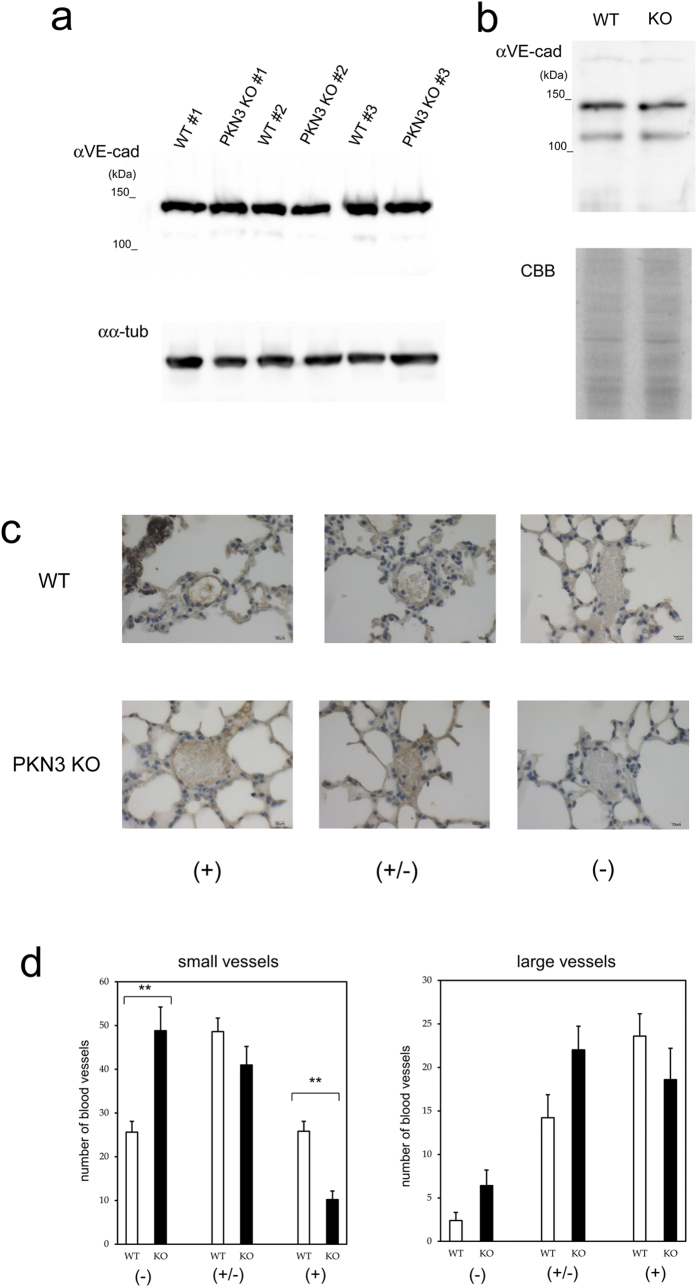
Expression of VE-cadherin in WT and PKN3 KO mouse lungs. (**a**) Immunoblotting of the crude extract of mouse lungs with anti VE-cadherin antibody. Each crude extract of lung from individual WT mouse (#1 – #3) and PKN3 KO mouse (#1 – #3) was prepared by homogenizing lung directly with sample buffer for SDS-PAGE. “αVE-cad” and “αα-tub” indicate immunoblotting with anti VE-cadherin and anti α-tubulin antibodies, respectively. (**b**) Immunoblotting of the plasma membrane fraction of mouse lungs with anti VE-cadherin antibody. The plasma membrane fractions of mouse lungs were prepared using Minute Plasma Membrane Protein Isolation Kit (Invent Biotechnologies, Inc.). “αVE-cad” indicates immunoblotting with anti VE-cadherin antibody. “CBB” indicates Coomassie staining of the gel after SDS-PAGE. (**c**) Immunohistochemical staining of mouse lungs with anti VE-cadherin. Blood vessels were classified into dense (indicated by + ), moderate (indicated by +/−), and weak (indicated by −) immunoreactive staining groups. (−) indicates no immunoreactivity or indistinguishable from red blood cells. (+/−) indicates mild immunoreactivity detectable only at x 400 magnification. (+) indicates obvious immunoreactivity detectable even at x 100 magnification. (**d**) The number of blood vessels classified by the VE-cadherin immunoreactivity. Blood vessels were first classified into two groups by size (“small vessels” and “large vessels”), and further classified by VE-cadherin immunoreactivity as described in (C). “small vessels” indicate thin vessels in the peripheral in the intra or inter alveolar area. “large vessels” indicate thick vessels rich in tunica media along bronchus or vessels radiating from that thick vessel to periphery. “small vessels” were counted up to 100/each slice of lung, and all of the “large vessels” were counted in each slice. ** indicates P < 0.01.

Santel *et al.* presented a hypothesis that the knockdown of PKN3 by RNAi promotes the elevation of VE-cadherin, a major protein involved in adherens junction integrity in the vascular endothelial cells, and prevents tumor metastasis through enhancement of this endothelial barrier^37^. Then we analyzed the expression level of VE-cadherin in the lungs of WT and PKN3 KO mice. Immunoblotting of mouse lungs with anti VE-cadherin antibody did not show significant difference in the amount of VE-cadherin between WT and PKN3 KO mice (Fig. 7a). Next, we biochemically isolated the plasma membrane fraction of lungs from the WT and PKN3 KO mice, and examined the expression of VE-cadherin by immunoblotting with anti VE-cadherin antibody. As shown in Fig. 7b, there was not a significant difference in the amount of VE-cadherin, either. Immunohistochemical staining of mouse lungs with anti VE-cadherin antibody revealed that the number of VE-cadherin-positive large blood vessels was not significantly different between PKN3 KO and WT mice, and that the VE-cadherin-positive small blood vessels were rather less in PKN3 KO mouse lung than WT (Fig. 7d).

### PKN3 siRNA impairs maturation of some cell-surface glycoproteins

**Figure 8:**
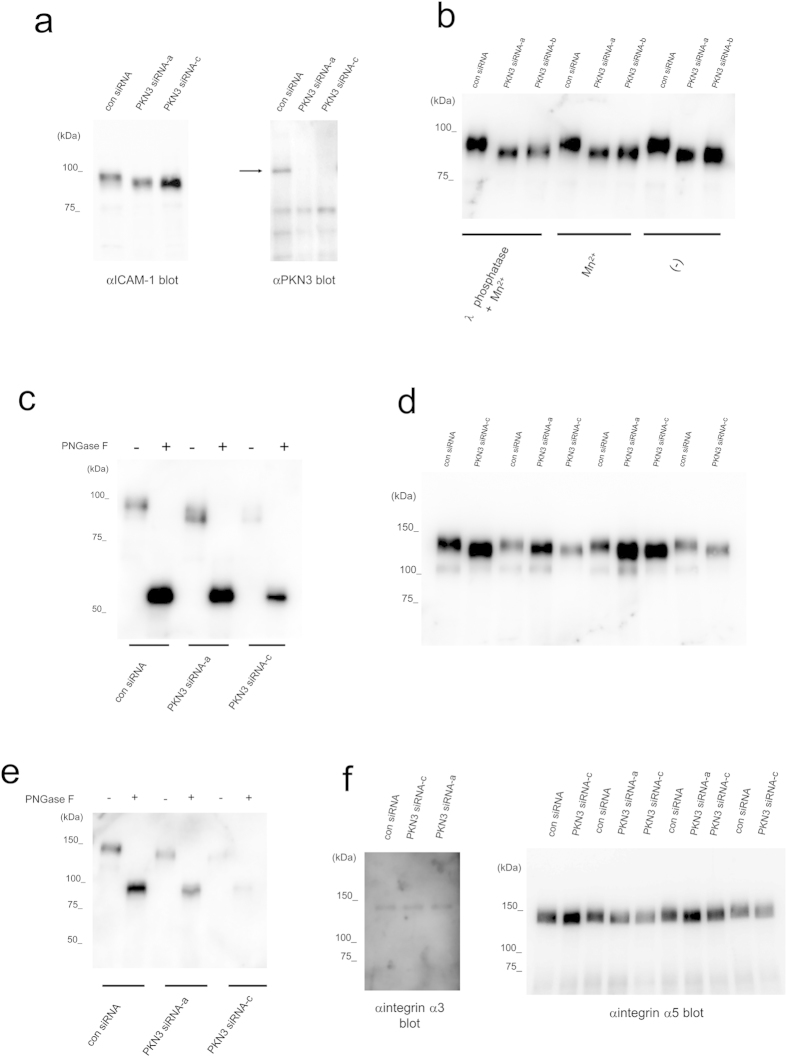
Immunoblotting of HUVECs with antibody against surface glycoproteins. “con siRNA” indicates validated universal negative control SIC-001 (Sigma-Aldrich Japan K.K.). “PKN3 siRNA-a” indicates Hs_PKN3_8290 (Sigma Aldrich Japan K.K.) derived from the coding sequence for ACC3 domain of human PKN3, “PKN3 siRNA-b” indicates Hs_PKN3_8291 (Sigma-Aldrich Japan K.K.) derived from the coding sequence for the catalytic domain of human PKN3, and PKN3 siRNA-c indicates M-004647-01 (Dharmacon Research) derived from the coding sequence for the catalytic domain of human PKN3. (**a**) Immunoblotting of HUVECs transfected with siRNA for PKN3 and control with antibody against ICAM-1 and PKN3. αNUS antibody was used for αPKN3 blotting. The arrow indicates the position of PKN3. (**b**) The effect of treatment of HUVEC extract with λ phosphatase on ICAM-1 immunoreactivity. (**c**) The effect of treatment of HUVEC extract with PNGase F on ICAM-1 immunoreactivity. (**d**) Immunoblotting of HUVECs transfected with siRNA for PKN3 and control with antibody against integrin β1. (**e**) The effect of treatment of HUVEC extract with PNGase F on integrin β1 immunoreactivity. (**f**) Immunoblotting of HUVECs transfected with siRNA for PKN3 and control with antibody against integrin α3 and α5.

Adhesive interactions of tumor cells with the endothelial cells are initial key events in the extravasation of tumor cells from the blood stream to the underlying tissue. Firm adhesion of tumor cells with the endothelial cells are mediated by cell adhesion molecules such as the Intercellular Adhesion Molecule-1 (ICAM-1) and Vascular Cell Adhesion Molecule-1 (VCAM-1) from the immunoglobulin family as well as the integrin family, leading to tumor invasion^38^,^39^. Several clinical and experimental studies show that high ICAM-1 activity is associated with tumor or metastasis progression whereas ICAM-1 depletion leads to reduced metastasis formation^40^,^41^,^42^,^43^,^44^. In order to examine the role of PKN3 on the adhesion molecules of vascular endothelial cells, PKN3 was knocked down using siRNA in HUVECs. HUVECs transfected with mock or with non-target siRNA contained two forms of immunoreactive ICAM-1 separated by SDS-PAGE, the ~95 kDa band of ICAM-1 with a less intense ~75 kDa band of ICAM-1 below (Fig. 8a). The ~95 kDa ICAM-1 immunoreactivity migrated faster from cells treated with PKN3 siRNA than that from control cells (Fig. 8a). Pretreatment with λ phosphatase before loading onto SDS-PAGE did not change the migration of the ICAM-1 immunoreactive band, suggesting that the phosphorylation defect induced by knockdown of PKN3 was not the major cause of this band shift (Fig. 8b). To ascertain whether the aberrant ICAM-1 seen after PKN3 siRNA treatment reflected impaired N-glycosylation, lysates of HUVECs were digested with peptide N-glycosidase F (PNGase F) before immunoblot analyses. PNGase F is an amidase that removes all saccharide moieties. PNGase F digestion completely converted the ~95 kDa ICAM-1 to ~53 kDa species both in PKN3 siRNA treated cells and control cells, which equates to the predicted molecular mass of ICAM-1 based on its primary aa sequence (Fig. 8c). These results suggest that the faster migration of ICAM-1 immunoreactive band from cells treated with PKN3 siRNA is caused by a defect of glycosylation of ICAM-1. Next we examined whether PKN3 depletion affects integrin family cell adhesion molecules. HUVECs transfected with non-target siRNA contained two forms of immunoreactive integrin β1 separated by SDS-PAGE, the ~130 kDa band of mature integrin β1 with a less intense ~110 kDa band precursor of integrin β1 below (Fig. 8d). Integrin β1 from cells treated with PKN3 siRNA migrated faster than that from the control cells in the ~130-kDa glycosylated β1 band, although the amount of integrin β1 did not differ significantly (Fig. 8d). PNGase F digestion completely converted the ~130-kDa integrin β1 to a band of 85 kDa (the size of the core protein) (Fig. 8e). Like ICAM-1 and integrin β1, integrin α5, but not integrin α3, from cells treated with PKN3 siRNA migrated slightly faster than that from the control cells. (Fig. 8f). These results suggest that PKN3 might be involved in the maturation of some endothelial cell-surface adhesion molecules such as ICAM-1, integrin β1 and α5.

We do not know the biochemical nature of this effect of PKN3 deficiency on the glycosylation of these endothelial cell-surface proteins. In general, N-linked protein glycosylation takes place in two distinct cellular compartments: the endoplasmic reticulum (ER) and the Golgi apparatus. ER stress seems not to be the main cause of altered ICAM-1 N-glycan composition by knockdown of PKN3, since the ~75kDa band of ICAM-1, bearing exclusively high-mannose N-glycan structures^45^, was not affected by introduction of PKN3 siRNA in our experiment (Fig. 8a–c), although ~95 kDa band of ICAM-1, bearing complex N-glycan structures^45^, was down-shifted (Fig. 8a–c). In the Golgi, N-glycans are known to be processed by multiple glycosyltransferases. Among them, Golgi N-acetylglucosaminyltransferase V (GnT-V) and III (GnT-III) are two major glycosyltransferases. GnT-V promotes the synthesis of a β1,6-branching GlcNAc structure. However, GnT-III catalyzes the introduction of a bisecting β1,4-GlcNAc residue that inhibits further processing and elongation of the N-glycans catalyzed by other glycosyltransferases including GnT-V^46^. In the case of ICAM-1, the N-glycan composition is reported to be modulated by all-trans-retinoic acid by upregulating GnT-III and down-regulating GnT-V via activation of the ERK signaling pathway, which causes a significant gel mobility down-shift of the ~95 kDa band of ICAM-1^47^. Integrin β1 is also reported to be a target of GnT-III^48^ and GnT-V^49^, and integrin α5 is shown to be a target of GnT-III^48^. Thus, PKN3 knockdown might inhibit ICAM-1, integrin β1 and α5 processing by activation of GnT-III or blocking of GnT-V or other glycosyltansferases. PKN3 could be involved in the regulation of gene expression of these glycosyltransferases, since PKN family members have been reported to work as regulators for transcription activity^1^,^50^,^51^,^52^. Another potential mechanism proposes the phosphorylation of the glycosylating enzymes by PKN3 and modulating their trafficking and substrate access, although most of them are type II transmembrane proteins and have a short cytoplasmic tail.

Notably, no obvious down-shift of migration of ICAM-1 immunoreactivity on SDS-PAGE for lung tissue extract from PKN3 KO mice was observed (Supplementary Fig. 4) compared to that of wild type mice. The possible reasons for the difference of the migration-shift of ICAM-1 on SDS-PAGE between the human cultured cells knocked-down of PKN3 and the organ tissues of PKN3 KO mice can be described as follows: i) Multiple glycosylation of mouse ICAM-1 masks the migration shift of this protein on the gel (Mouse ICAM-1 shares five consensus N-glycosylation sites with human ICAM-1, but comprises additional five N-glycosylation sites in the first and fifth Ig domains^53^). ii) Lung tissue contains many types of cells other than vascular endothelial cells such as fibroblasts and macrophages, showing the complexity and heterogeneity of glycan formation (ICAM-1 N-glycan content depends on the cell type in which the protein is produced^54^). The above seeming discrepancy between the cultured cells and the organ tissues may also raise the classical question as to whether cultured cells are a reliable source for investigating surface glycosylation changes. However, use of such *in vitro* culture models has given some potential insights into the regulation of cell surface glycan expression in previous studies^55^,^56^. Therefore, the changes of glycosylation of cell surface adhesion molecules should be added to the candidate mechanisms induced by PKN3 KO, providing directions to guide further work.

## Discussion

This paper represents the first description on the role of PKN3 in angiogenesis and tumor metastasis by establishing PKN3 KO mice. We demonstrated that genetic ablation of PKN3 inhibits angiogenesis *ex vivo* (aortic ring assay) and *in vivo* (corneal micropocket assay) and gives resistance to lung metastasis after tail vein injection of melanoma.

What is the role of PKN3 in angiogenesis? Angiogenesis requires complex interactions of signals and physical forces orchestrating the activities of endothelial cells, pericytes, fibroblasts, and smooth muscle cells^31^. Aleku *et al.* reported that treatment of HUVECs with Atu027, an siRNA-lipoplex directed against PKN3, impaired tube formation on matrigel and migration activity in Boyden Chamber assay and scratch assay^30^. Our data showed that ablation of the PKN3 gene suppressed migration of embryonic fibroblast cells induced by various growth factors (Fig. 2). Considering these data, PKN3 is required for efficient angiogenesis, presumably by modulating cell motility of angiogenesis-related cells, such as endothelial cells and fibroblastic cells at the actin-cytoskeleton level. However, our data showed that PKN3 ablation had no impact on tumor angiogenesis, consistent with the previous report describing the orthotopic cancer model treated with Atu027 evaluated by CD31 or CD34 immunoreactivity^30^. So, why does PKN3 appear not necessary during tumor angiogenesis? One possible explanation would be that other PKN isoforms could compensate for the loss of PKN3 function in the context of tumor. PKN1, in particular, might be stimulated in the stromal cells inside of a tumor, because ischemic/hypoxic stress, which frequently happens inside of a tumor, is reported to activate or enhance the expression of PKN1^57^,^58^,^59^,^60^.

To obtain information on the possible target point of PKN3 in angiogenesis, we next focused on the glycosylation events of several important cell surface proteins involved in angiogenesis such as integrin, because the initiation of angiogenesis by proangiogenic growth factors depends on interactions between the endothelium and various extracellular matrix proteins mediated by integrins^61^. The β1 integrin in endothelial cells is known to participate in angiogenesis with various partner α integrins, and α5β1 integrin binds fibronectin, contributing to an angiogenesis pathways^62^. Among the integrins we examined, integrin β1 and α5 showed faster migration in SDS-PAGE in HUVECs transfected with PKN3 siRNA, suggesting a glycosylation defect of these proteins in HUVECs knocked-down of PKN3 (Fig. 8d,f). The alterations in the oligosaccharide portion of integrins are reported to regulate cell phenotypes^63^,^64^. The function of β1 integrin depends on its accurate glycosylation. Actually, N-glycosylation of integrin α5β1 plays a crucial role in cell spreading, cell migration, ligand binding, and dimer formation^65^. Therefore, poor angiogenesis in PKN3 KO mice might be explained by the alteration of glycosylation of these integrins.

The next important question would be the cause of the reduction of metastatic foci in the PKN3 KO mouse lung. Leenders *et al.* demonstrated that induced knockdown of PKN3 expression in PC-3 prostate cancer cells by shRNA interferes with formation of lymph node metastasis in an orthotopic mouse prostate tumor model, suggesting that PKN3 in tumor cells may primarily be required for metastatic growth of these cells^25^. Here, we present *in vivo* genetic evidence that PKN3 in stromal cells play important roles in metastasis as evidenced by significantly smaller number and the smaller size of the metastatic tumor foci in the lungs of PKN3 KO mice than WT mice (Fig. 6). The potential difference of angiogenic activity between WT and PKN3 KO mice does not seem to cause this difference, because the tumor diameters were overall less than 1 mm at the time point of mouse dissection (14th day after the tumor injection), and the PKN3 KO mice did not show suppression of tumor angiogenesis from our subcutaneous tumor implant experiments (Fig. 5). The difference in number and size of the metastatic tumors may arise from the substantial disturbance of tumor cell extravasation in PKN3 KO mice than in WT control. If so, what is the potential mechanism of the block of tumor cell extravasation in PKN3 KO mice? Santel *et al.* reported that HUVECs knocked down of PKN3 by siRNA revealed elevated levels of VE-cadherin by immunoblotting and confocal microscopy^37^. Furthermore, Atu027-treated mice showed the elevated expression of VE-cadherin in pulmonary blood vessels in lung, suggesting that the reduction of the intravasation and extravasation of tumor cells by increasing the endothelial adherens junction composed of VE-cadherin^37^. Notably, however, our immunoblotting and immunohistochemical analysis (Fig. 7) did not reveal the overexpression of VE-cadherin in PKN3 KO mouse lung tissues, even with clear metastatic foci of melanoma. We also evaluated the vascular permeability in the Lewis lung cancer tissues reported in Fig. 5 by using the immunoreactivity of fibrinogen/fibrin around blood vessels as an endogenous indicator^66^,^67^,^68^ (Supplementary Fig. 5). This experiment showed that the vascular permeability of PKN3 KO mice was not significantly different from that of WT mice. Together with our data about VE-cadherin expression levels (Fig. 7), the impairment of lung metastasis of melanoma in PKN3 KO mice may not be simply due to the stable elevation of the endothelial barrier in PKN3 KO mice. The reversible endothelial cell retraction is an important process in tumor cell extravasation^69^, therefore the mobility defect of the stromal cells such as endothelial cells in PKN3 KO mice might inhibit the tumor cell extravasation. Additional feature observed in HUVECs knocked down of PKN3 was defective glycosylation of ICAM-1 (Fig. 8), a central adhesion molecule important for binding and signaling between vascular endothelial cells and tumor cells during the tumor metastasis. The various functions of ICAM-1 appear to be differentially regulated by N-linked glycosylation^70^. Furthermore, it has been recently reported that glycosylation deficiency of ICAM-1 hinders cell adhesion and trans-endothelial migration of hematological cells^47^,^71^. Therefore, the glycosylation defect of ICAM-1 might be involved in the inhibition of tumor cell extravasation in PKN3 KO mice.

What is the immediate upstream regulator of PKN3 in angiogenesis and tumor metastasis? PKN3 has been reported to bind to RhoA, RhoB, and RhoC small GTPases, showing preferred association with RhoC^14^. We also confirmed that PKN3 selectively binds to the GTP-bound form of RhoC, but not to the GDP bound form or the effector domain mutant of RhoC (Supplementary Fig. 6), whereas Hutchinson *et al.* reported that PKN3 are likely targets for RhoB rather than RhoA or RhoC on the basis of affinity by direct binding experiments^13^. Notably, RhoC is reported to be essential for angiogenesis and the downstream regulator of VEGF in endothelial cells by a matrigel plug assay using nude mice treated with RhoC-RNAi retrovirus and a tube formation assay of a human dermal microvascular endothelial cell line (HMEC-1) in three-dimensional matrigel^72^. Hakem *et al.* generated RhoC KO mice and demonstrated that the loss of RhoC does not affect tumor development but drastically inhibits tumor metastasis, whereas RhoC was absent both in the host and in the tumor cells in their mouse model^73^. Intriguingly, they also showed that deficiency of RhoC does not affect tumor angiogenesis by investigating the levels of CD31 and Factor VIII expression in primary tumors in their mouse model^73^. Assuming that PKN3 is the direct target of RhoC in stromal cells, the above reports seem to be consistent with our data showing that PKN3 is involved in potential angiogenic activity and that PKN3 KO in stromal cells inhibits metastasis but not primary tumor growth and *in vivo* tumor angiogenesis.

The shRNA knockdown of PKN3 in an orthotopic PC-3 prostate cancer model had a profound effect on primary tumor growth^14^, whereas an earlier report had demonstrated the relatively small effect on primary tumor growth using the same cancer cell line^25^. Thus it is still promising that in some conditions PKN3 blocker suppresses the primary cancer growth in a cell autonomous-fashion. With regard to the metastasis of cancer, previous studies^14^,^25^,^74^ consistently support the suppressive effect of the PKN3 blocker on the metastasis in a cell autonomous-fashion, and our study also confirmed the suppressive effect in a non-cell autonomous fashion. Therefore, it is conceivable that PKN3 blocker would target both tumor cells and stromal cells when used as a potential cancer therapy.

We have demonstrated, the critical role of PKN3 in stromal cells in regulation of tumor metastasis. The expression profile of stromal PKN3 might act as a prognostic marker of cancer. Our results also indicate PKN3 as a possible regulator of neovascularisation and provide a novel insight into the molecular control of angiogenesis. We speculate that PKN3 blocking strategies not only could be applied in cancer treatment, but also for other vascular diseases such as arthritis and age-related macular degeneration^75^.

## Methods

### Generation of PKN3 KO mice

A genomic fragment of the mouse PKN3 gene was isolated from mouse BAC library RPCI-23 (C57BL/6). For disruption of PKN3, a replacement-type targeting vector was first made (Fig. 1a). It contained a ~8 kbp *Sal*I–*Not*I DNA fragment including exon 3-exon 16 of PKN3, neomycin selection cassette, and a ~4 kbp *Xba*I/*Nhe*I DNA fragment including exon 20-exon 22 and followed by the diphtheria toxin (DT) gene for negative selection. TT2 embryonic stem (ES) cells were transfected with the linearized targeting vector by electroporation^76^. Two of the 192 G418-resistant ES cell clones carried a correctly targeted PKN3 allele, as assessed by PCR analysis and Southern blot analysis of genomic DNA. The ES clone was injected into ICR 8-cell stage embryos for generation of chimeric mice. A high percentage male chimeric mice, as judged by the agouti coat color, were mated with C57BL/6 mice to determine germline transmission. F1 mice were crossed with EIIa-*cre* transgenic mice^77^ to remove neomycin selection cassette, and were backcrossed at least 16 times into the Charles River C57BL/6N background before phenotypic analysis. PKN3 KO mice carrying the homozygous deletion (PKN3 −/−) were viable, born at a frequency expected for Mendelian inheritance. PKN3 mouse line has been registered as Accession No. CDB0457K: http://www.clst.riken.jp/arg/mutant%20mice%20list.html.

### Genotyping and preparation of antibodies

See method section of Supplementary Information.

### Animals

This study was approved by the Kobe University and Kinki University Animal Care and Use Committees and carried out according to the institutional animal experimentation regulations.

### Cell culture and siRNA transfection

B16 melanoma BL6 cells (B16BL6 cells) were supplied by Dr. Inufusa (Kinki University, Osaka, Japan) and cultured in RPMI 1640 medium (Sigma) supplemented with 10% fetal calf serum (FCS) (Gibco, Carlsbad, CA, USA), 100 μg/ml penicillin (Gibco), 100 U/ml streptomycin (Gibco), and 25 mM HEPES (pH 7.4; Wako, Tokyo, Japan) in an atmosphere containing 5% CO_2_. Lewis lung cancer cells were obtained from Riken Cell Bank (Ibaraki, Japan), and cultured in Dulbecco’s Modified Eagle’s medium (Sigma, St. Louis, MO, USA) supplemented with 10% FCS (Gibco), 100 μg/ml penicillin (Gibco), 100 U/ml streptomycin (Gibco), and 25 mM HEPES (pH 7.4; Wako, Tokyo, Japan) in an atmosphere containing 5% CO2. HUVECs were purchased from Lonza. HUVECs were cultured in EC growth medium (EGM-2 BulletKit, Lonza) supplemented with 2% fetal bovine serum (FBS). For RNA-mediated interference of PKN3 expression, the following siRNAs were used: Hs_PKN3_8290 (Sigma-Aldrich Japan K.K.), Hs_PKN3_8291 (Sigma-Aldrich Japan K.K.), and siGENOME SMARTpool Human PKN3 M-004647-01 (Dharmacon Research). SIC-001, a validated universal negative control provided by Sigma-Aldrich Japan K.K., was used as the negative control siRNAs that do not target any mammalian gene. Cells cultured in a 6-well plate were transfected with 10 nM siRNA with Lipofectamine RNAiMAX (Invitrogen) or Lipofectamine 3000 (Invitrogen). Similar results were obtained in either case of using Lipofectamine RNAiMAX (Invitrogen) or Lipofectamine 3000. For immunoblot analysis, cells were lysed by scraping into SDS-sample buffer.

Mouse embryonic fibroblasts derived from E14 embryos were prepared according to standard procedures, and maintained in DMEM.

### Immunoblot analysis

Samples were subjected to 6%–10% SDS-PAGE and separated products were subsequently transferred to a polyvinylidene difluoride membrane. The membrane was then blocked with TBS (20 mM Tris/HCl at pH 7.5, 137 mM NaCl) containing 0.05% Triton X-100 (TBS-T) and 5% normal goat serum or Blocking One (nacalai tesque, Japan) for 1 hr at room temperature. The membrane was then incubated in TBS-T and the primary antibody for 1 hr at room temperature or for O/N at 4 °C. The membrane was washed three times (5 min each time) in TBS-T before incubating the blot in TBS-T containing the secondary antibody conjugated to horseradish peroxidase at 1:2000–1:10000 dilution for 45 min. After this incubation, the membrane was subjected to three 10 min washes in TBS-T. Blots were developed by the enhanced chemiluminescence method.

### Quantification of PKNs

Mouse tissues were removed quickly after decapitation and added to 9 vol of 50 mM Tris/HCl, pH 7.5, containing 5 mM EDTA, 5 mM EGTA, 0.5 mM dithiothreitol (DTT), 10 μg/ml leupeptin, and 1 mM phenylmethylsulfonylfluoride (PMSF), and homogenized with 10 strokes of a Teflon/glass homogenizer. The crude lysates of various tissues were used for quantification of PKNs by densitometric analysis of immunoreactivity of PKNs on Western blotting using αC6 antibody (for PKN1), αParN2 antibody (for PKN2), and αNUS antibody (for PKN3) as described previously^28^. Various amounts (1–10 ng) of the purified GST-fused C-terminal 310 aa region of mouse PKN1, GST-fused N-terminal 506 aa region of mouse PKN2, and GST-fused N-terminal 125 aa region of mouse PKN3 were used as standards of quantification for PKN1, PKN2, and PKN3, respectively.

### Immunohistochemistry

The lungs and isolated tumors were fixed in 4% paraformaldehyde buffered with PBS (pH 7.2) and embedded in paraffin. Four-micrometer-thick sections were stained with primary antibodies as indicated in each figure, and the immune complexes were visualized by HISTOFINE Simple Stein Mouse MAX-PO kit suitable for individual primary antibody (Nichirei Corporation, Tokyo) and 3-3′ Diaminobezidine-4HCl (DAB) according to the manufacturers’ instructions.

### *In vitro* migration assay

Migration was analyzed by Boyden chamber assay using Transwell cell culture inserts (8.0 μm pore size; Costar, Cambridge, MA). Adjusted viable cell concentration was counted with trypan blue exclusion. Mouse embryonic fibroblasts were plated (5 × 10^5^ cells/ml in serum-free DMEM) in the upper chamber. The lower chamber containing various growth factors indicated in the figure legend. After 5 h, the cells remaining on the upper surface of the membrane were wiped off, and cells migrating to the lower surface in triplicate wells were visualized with Diff-Quick stain (Sysmex, Japan) and counted in each of four randomly chosen light microscopic fields at × 20 objective.

### *In vivo* Tumor Growth Study

Freshly cultured Lewis lung cancer cells resuspended in sterile 1 × PBS and 1 × 10^6^ cells per 100 μl were injected subcutaneously between the shoulder blades of approximately 10-week-old female WT and PKN3 KO mice (six mice per condition). Tumors were measured every 5 days using Vernier calipers and volume calculated (length × width^2^ × 0.52). After 30 days, tumors were collected, and were fixed in 4% paraformaldehyde for histologic analysis.

### Experimental metastasis of tumor cells

B16 BL6 cells (1 × 10^5^ cells in 0.2 ml) were injected into the tail vein of 10-week-old female WT and PKN3 KO mice after viable cells were counted with trypan blue exclusion. The mice were anesthetized with pentobarbital and sacrificed at 14 d after the cell injection. Subsequently, their lungs were excised and fixed in a paraformaldehyde solution. Nodules visible from outside as black forms in the lungs were then enumerated. Then the 10 step slice sections with 20 μm intervals were prepared from each mouse lung and were stained with anti S100p antibody, and the analyzed numbers and areas of tumors were measured using image analysis software (Motic Image plus 2.5; SHIMADZU, Kyoto, Japan).

### Aortic ring assay

Aortic ring assay was carried out as described in^78^. Briefly, thoracic aortas were isolated from WT and PKN3 KO mice under a dissecting microscope, cut into 1-mm sections, and embedded in 24-well plate collagen (Nitta gelatin type I-A) or matrigel (Becton Dickinson Biosciences) -coated plates. Medium containing 2% fetal bovine serum and 30 ng/ml VEGF (VEGF164, ORFgen) or 25 ng/ml PDGF (Peprotech) or 20 ng/ml HGF (Peprotech) or 30 ng/ml bFGF (Peprotech) was added to each well of gelled collagen (in case of VEGF, HGF, and bFGF) or matrigel (in case of PDGF). The plates were incubated at 37 °C in a 5% CO_2_ incubator. Microvessel-like properties of sprouting structures were observed under microscope.

### Corneal micropocket angiogenesis assay

Corneal angiogenesis was assessed as described^33^. Briefly, corneal micropockets were created using a Mani Ophthalmic Knife in one side eye of 9–10 week-old mice. Micropellets containing 80 ng bFGF were implanted into each corneal pocket at 0.8 mm from the limbus. After 7 days, the eye was viewed under a dissecting microscope. Finally, vessel area (VA, mm^2^) was calculated using the formula VA = 0.02 π × VL × CH, where VL is the vessel length (in tenths of millimeters) and CH is the clock hours^33^.

### Preparation of HUVEC extract and phosphatase and glycosidase treatment

For λ phosphatase treatment, HUVECs from one well of six-well plates were lysed in 100 μl of cold lysis buffer (50 mM Tris/HCl at pH 7.5, 100 mM NaCl, 0.1% Brij 35, 5 mM EGTA, 2 mM DTT, 1 mM PMSF, 10 μg/ml leupeptin) and homogenized using 27 Gauge syringe. After centrifugation at 4 °C at 13,000 × g for 15 min, 30 μl of the supernatant was incubated at 30 °C for 1 hr with/without 2 mM MnCl_2_ and λ phosphatase (New England Biolabs) as indicated in the figure. The resultant sample was subjected to immunoblotting. For PNGase F treatment, HUVECs from one well of six-well plates were lysed in 100 μl of cold lysis buffer (50 mM Tris/HCl at pH 7.5, 150 mM NaCl, 1 mM DTT, 1 mM EDTA, 0.1% Triton X-100, 5 μg/ml leupeptin) and homogenized using 27 Gauge syringe. After centrifugation at 13,000 × g for 15 min at 4 °C, 5 μl of the supernatant was incubated with 1 μl of 10 × glycoprotein denaturing buffer (New England Biolabs) and 4 μl of H_2_O at 95 °C for 5 min, then chilled on ice. This total reaction was mixed with 2 μl of 10 × G7 reaction buffer, 2 μl of 10% NP-40, and 6 μl of H_2_O, and with/without 1 μl of PNGase F (New England Biolabs) as indicated in the figure. The resultant sample was subjected to immunoblotting.

### Statistical analysis

All experiments were performed independently in at least triplicate and a Student’s t-test was used to examine the differences between the two groups of data. Differences with p < 0.05 were considered statistically significant.

## Additional Information

**How to cite this article**: Mukai, H. *et al.* PKN3 is the major regulator of angiogenesis and tumor metastasis in mice. *Sci. Rep.* **6**, 18979; doi: 10.1038/srep18979 (2016).

## Supplementary Material

Supplementary Information
