# Supplementary material for: PKN3 is the major regulator of angiogenesis and tumor metastasis in mice
Source: Sci Rep. 2016 Jan 8;6:18979. doi: 10.1038/srep18979 (PMC4705536; doi:10.1038/srep18979)
Supplement: Supplementary Information [file srep18979-s1.pdf]

SUPPLEMENTARY INFORMATION

PKN3 is the major regulator of angiogenesis and tumor metastasis in mice

Hideyuki Mukai[1*], Aiko Muramatsu[2], Rana Mashud[3], Koji Kubouchi[4], Sho Tsujimoto[4], Hongu Tsunaki[5], Yasunori Kanaho[5], Masanobu Tsubaki[6], Shozo Nishida[6], Go Shioi[7], Sally Danno[3], Mona Meherba[3], Ryosuke Satoh[4], and Reiko Sugiura[4]

[1]Biosignal Research Center, Kobe University, Kobe 657-8501, Japan

[2]Graduate School of Science and Technology, Kobe University, Kobe 657-8501, Japan

[3]Graduate School of Medicine, Kobe University, Kobe 657-8501, Japan

[4]Laboratory of Molecular Pharmacogenomics, School of Pharmaceutical Sciences, Kinki University, 3-4-1 Kowakae, Higashi-Osaka 577-8502, Japan

[5]Graduate School of Comprehensive Human Sciences, Institute of Basic Medical Sciences, University of Tsukuba, Ibaraki 305-8575, Japan

[6]Division of Pharmacotherapy, Kinki University School of Pharmacy, Kowakae, Higashi-Osaka 577-8502, Japan.

[7]Genetic Engineering Team, Division of Bio-function Dynamics Imaging, RIKEN Center for Life Science Technologies (CLST), 2-2-3 Minatojima Minami,Chuou-ku, Kobe 650-0047

* to whom correspondence should be addressed (mukinase@kobe-u.ac.jp).

Supplementary methods

Genotyping

Genomic DNA was isolated from ES cells and mouse tail snips by standard techniques and subjected to Southern blot analysis and PCR analysis for identification. Screening of ES cells was performed through genomic PCR using primers: N3-R2 (5'-- GAAGAGGGTCATCAGGTGGAAGTCAG--3') and L-Neo1 (5'--GTACTCGGATGGAAGCCGGTCTTGTC--3') to yield PCR products of ~5 kbp for the mutant allele. N3-R2 primer matches the sequence located immediately just 3' to, but not contained within, the targeting vector, and the L-Neo1 primer matches the sequence located in the neo gene. Reaction conditions were as follows: 95°C for 1 min for 1 cycle, and 98°C for 20 sec, 68°C for 7 min for 45 cycles, and 72°C for 15 min for 1 cycle. Southern blot analysis was performed using genomic DNA digested with *Apa*I and *Bam*HI and probed with probe A and probe B as indicated in the Fig. 1A, respectively. WT (+) and mutant alleles (−) containing Neo cassettes are indicated by the presence of a 9.9 kbp (+) versus 12.4 kbp (−) ApaI for probing with probe A, and a 12.7 kbp (+) versus 10.1 kbp (−) *Bam*HI DNA fragment for probing with probe B (Fig. 1B). Genotyping of mouse tail, for discrimination of WT and mutant lacking Neo cassette, was performed using the PCR primers N3-LF7 (5'--GTGTGAGCCAGACTCTATCACTGAGC--3'), W-GF (5'--ATAACCAGAACTACGGTGTGTGTGCC--3'), and GL (5'--GGACACTGTGGAAATTGGGGTTCAG--3') , yielding PCR products of 298 bp (WT allele) and 430 bp (mutant allele) (Fig. 1C). Reaction conditions were as follows: 94°C for 5 min for 1 cycle, and 94°C for 1 min, 68°C for 1 min, 72°C for 1min for 35 cycles, and 72°C for 5 min for 1 cycle.

Antibodies

The polyclonal antibodies αC6 against PKN1 were prepared as described [1]. The polyclonal antiserum for αParN2 were prepared by immunizing rabbits with the bacterially-synthesized fragments of glutathione S transferase (GST) -fused N-terminal 506 amino acid (aa) of human PKN2. αParN2 antibodies were affinity-purified by CNBr-activated Sepharose 4B (Amersham Bioscience) conjugated with GST-fused N-terminal 491 aa of mouse PKN2. The polyclonal antiserum for αNUS was raised by immunizing rabbits with bacterially-synthesized fragments of GST-fused N-terminal 302 aa of human PKN3. αNUS antibodies were affinity-purified by CNBr-activated Sepharose 4B conjugated with GST-fused N-terminal 125 aa of mouse PKN3. Anti-S100 protein rabbit polyclonal antibody (#NCL-L-S100p) was purchased from Leica-Novocastra, Newcastle upon Tyne, UK. The anti-integrinβ1 rabbit polyclonal antibody (#4706), anti-integrin α5 rabbit polyclonal antibody (#4705), anti p38

rabbit polyclonal antibody (#9212), anti phospho-p38 (Thr180/Tyr182) rabbit monoclonal antibody (#4511), anti p44/42 (ERK1/2) rabbit polyclonal antibody (#9102), anti phospho-p44/42 (ERK1/2)(Thr202/Tyr204) rabbit polyclonal antibody (#9101), and anti α-tubulin mouse monoclonal (DM1A) antibody (#3873) were purchased from Cell Signaling Technology. The anti ICAM-1 mouse monoclonal (G-5) antibody and anti integrin α3 goat polyclonal antibody were purchased from Santa Cruz Biotechnology. The anti-CD31 (SZ31) rat monoclonal antibody (#DIA-310) was purchased from Dianova, Hamburg, Germany. The anti-VE-Cadherin rabbit polyclonal antibody was purchased from Abcam plc, Cambridge, UK. The anti-fibrinogen rabbit polyclonal antibody (#F0111) was purchased from DAKO. Alexa Fluor 594 goat anti rat IgG (#A11007) and Alexa Fluor 488 goat anti rabbit IgG (#A11034) were purchased from Life technologies.

Permeability experiment of endothelial cells

To visualize the amount of fibrinogen deposition around tumor blood vessels, sections of the Lewis lung cancer tissues grown subcutaneously in WT and PKN3 KO mice (as prepared in Fig. 5) were incubated with anti-fibrinogen and anti CD31 antibodies before incubation with Alexa 488–and Alexa 594–conjugated secondary antibodies. To quantify the amount of colocalization between fibrinogen and CD31, two areas per slide were scored from six tumors from PKN3 KO or WT mice, respectively. Each area was quantified using the Image J[2] and its colocalization module JACoP[3].

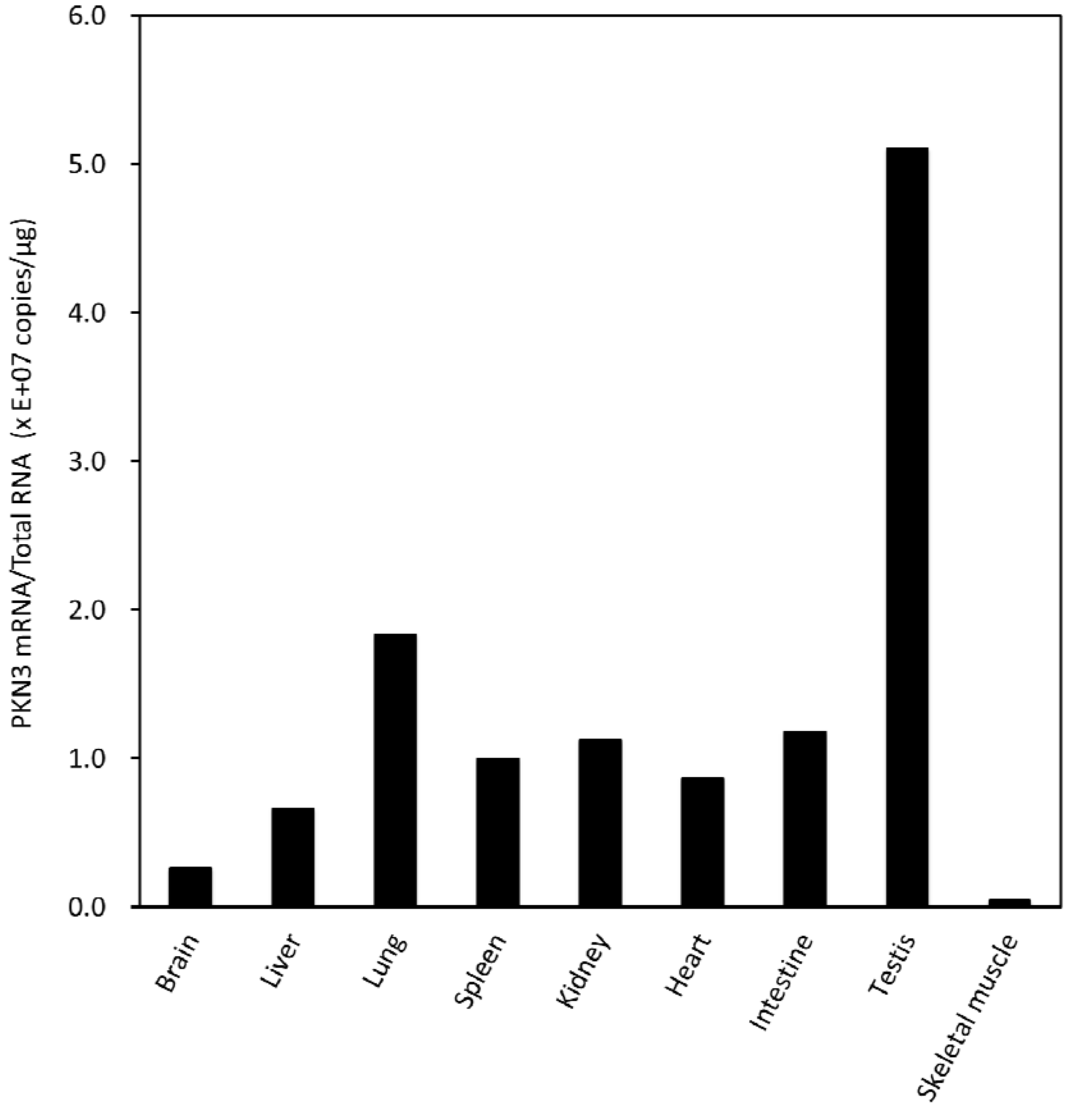

Supplementary Fig. 1. Quantification of PKN3 mRNA copies in 1 µg of total RNA

Total RNA was isolated from the tissue samples using RNeasy Total RNA Mini Kit (Qiagen). Quantitative real-time PCR was done using a standard Taqman PCR kit protocol on a LightCycler 480 (Roche Diagnostics, Penzberg, Germany). The 40 µL PCR reaction included 2 µL RT products, 0.8 µL Taqman mRNA assay primers (each 10 µM) and 0.4 µL UPL probe # 21 (10 µM), 20 µL LightCycler® 480 Probe Master; 2× conc. (Roche Diagnostics, Penzberg, Germany), and 16 µL water. The reactions were incubated in a 96-well plate at 95 °C for 10 min, followed by 45 cycles of 95 °C for 10 sec, 60 °C for 30 sec, and 72 °C for 1 sec. No amplification of the signal was observed when water was added instead of cDNA sample. A standard curve was constructed by plotting Cp values against the logarithmic concentration of the starting PKN mRNA with different concentrations. The amount of an unknown sample was quantified by interpolating the Cp values in the standard curve.

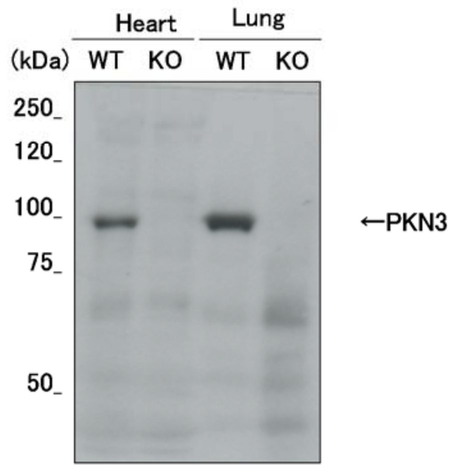

Supplementary Fig. 2. Immunoblotting of mouse tissues using antibody against the N-terminal of PKN3

Immunoblotting was performed using αNUS antibody.

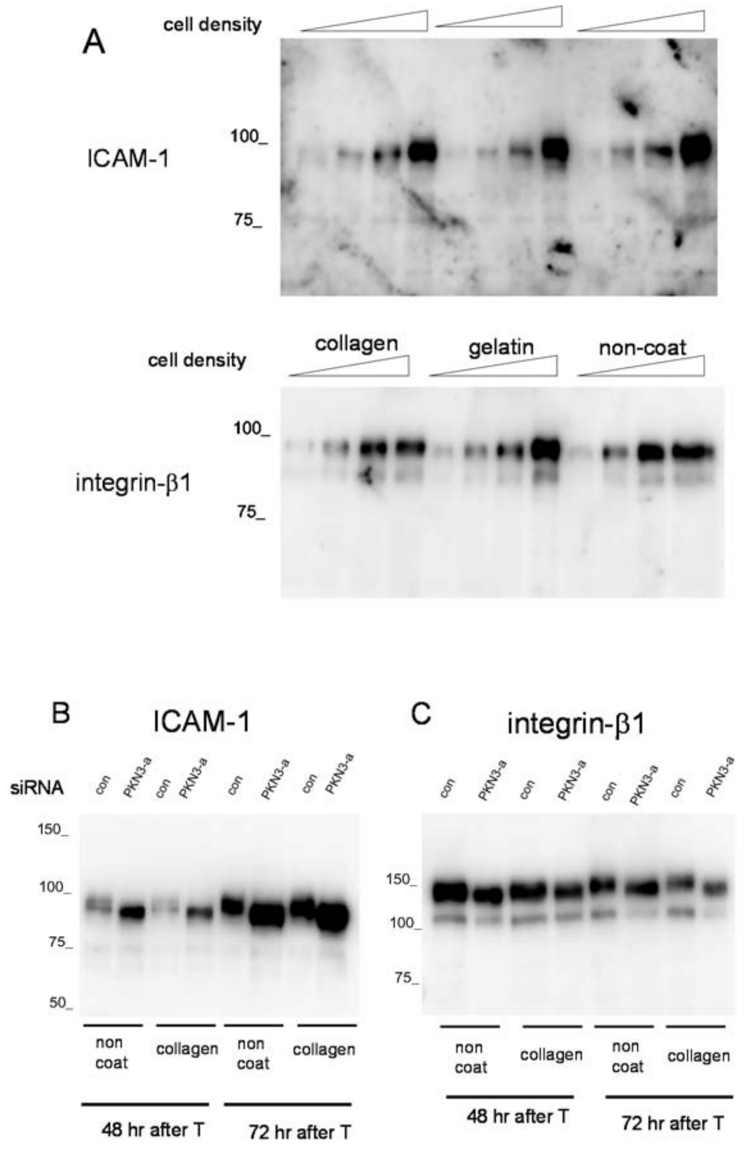

Supplementary Fig. 3. The effects of culture conditions on immunoblotting of HUVECs with anti ICAM-1 and integrin β1 antibody

(A) The effect of cell density of HUVECs on the migration of ICAM-1 and integrin β1 immunoreactivity.

The cell density was changed from 10% - 100% confluency on collagen, gelatin, and non-coated dishes. The cell extract was prepared by homogenizing cells using SDS-sample buffer. Neither the difference of the confluency of cultured cells nor coating of dishes affected the migration level of ICAM-1 and integrin β1.

(B and C) The effect of the incubation time after siRNA transfection on the migration of ICAM-1(B) and integrin β1(C) antibody.

The cells were maintained on collagen or non-coated dishes.

The cells were collected by homogenization using SDS-sample buffer at 48 hr and 72 hr after 10 nM siRNA transfection. In each condition, the faster migration of ICAM-1 and integrin β1 was observed in cells transfected with PKN3 siRNA.

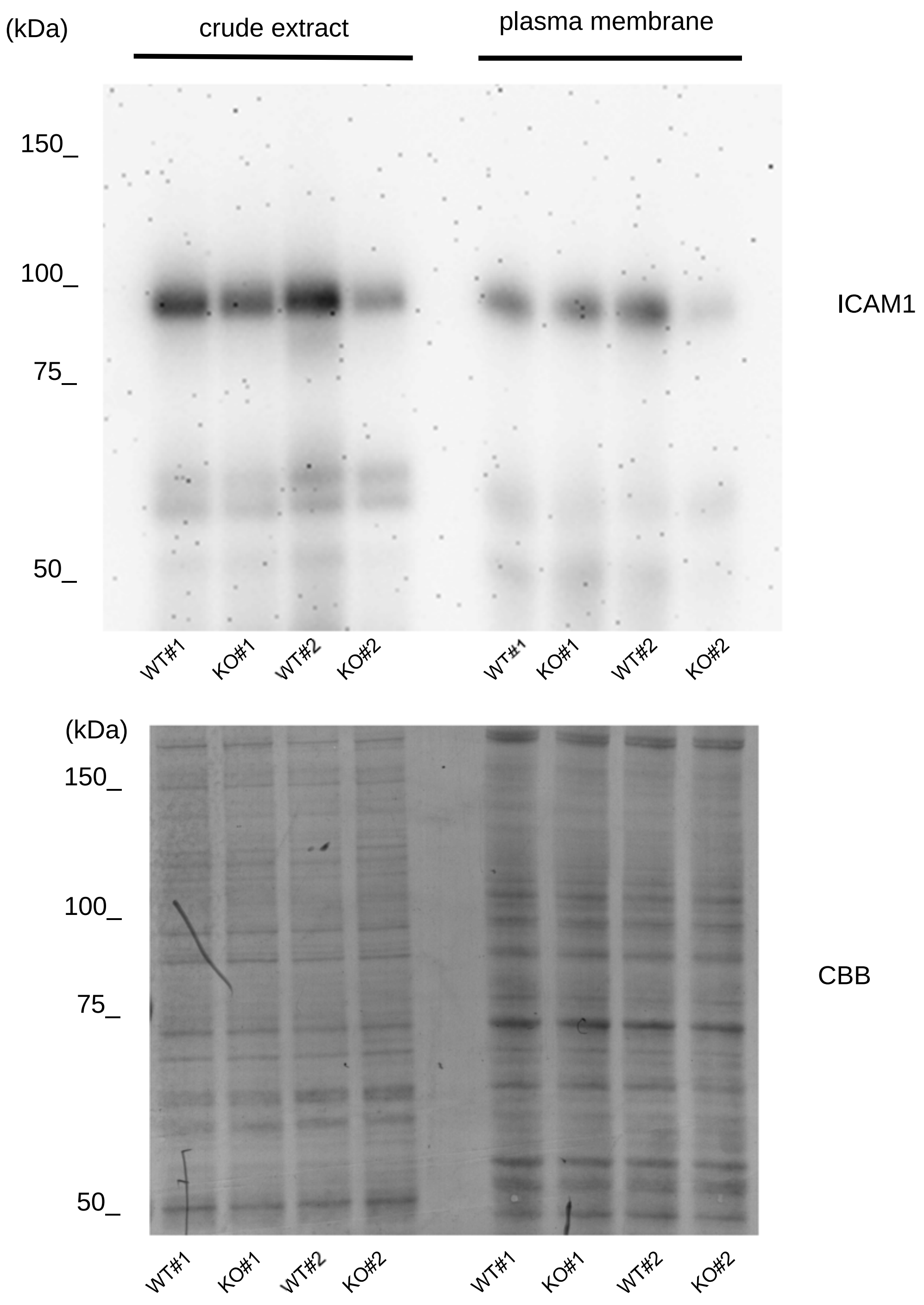

Supplementary Fig. 4. Immunoblotting of mouse lung extract using anti ICAM-1 antibody.

Crude lung fractions were prepared by homogenizing mouse lung in 9 vol of homogenizing buffer (50 mM Tris/HCl at pH 7.5, 100 mM NaCl, 5mM EGTA, 5 mM EDTA, 0.5 mM DTT, 1 mM PMSF, 1 μg/ml leupeptin) with Dounce homogenizer. The plasma membrane fractions of mouse lungs were prepared using Minute Plasma Membrane Protein Isolation Kit (Invent Biotechnologies, Inc.). The resultant sample was subjected to immunoblotting.

WT, wild type; KO, PKN3 KO; ICAM-1, Western blotting using anti ICAM1 antibody; CBB, Coomassie brilliant blue staining of the PVDF membrane

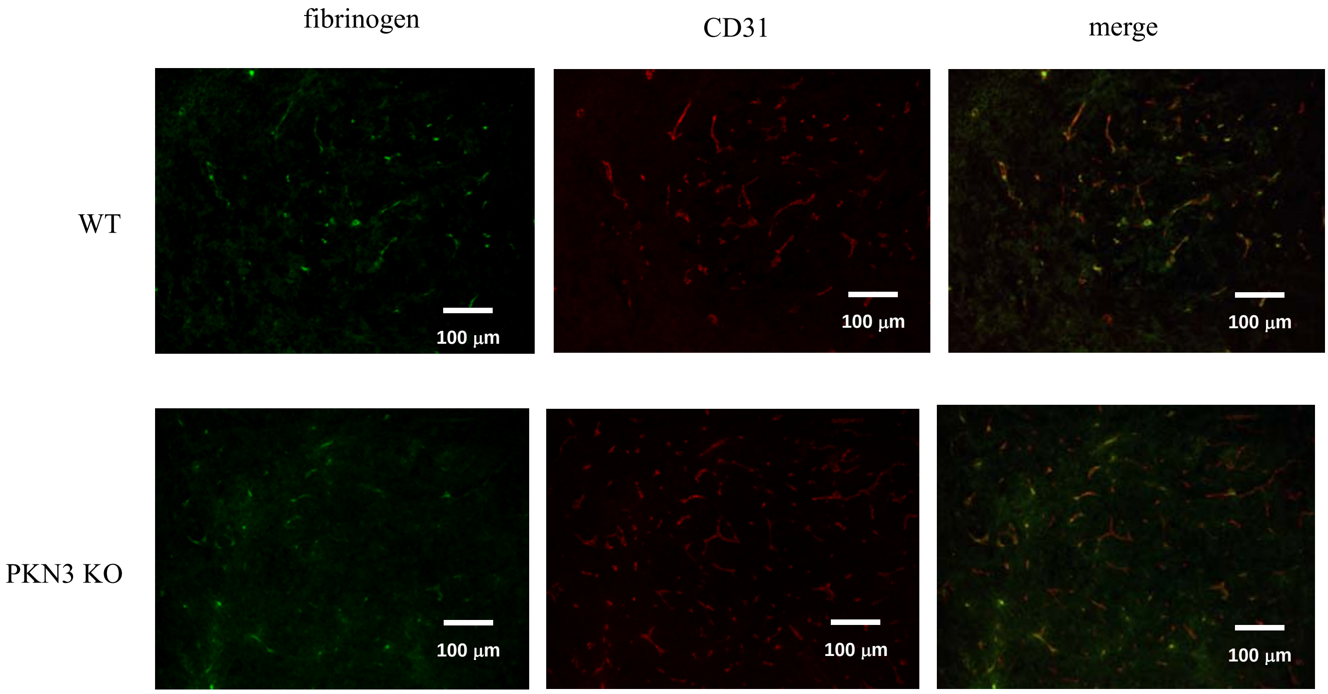

Supplementary Fig. 5. Fibrinogen and CD31 staining for plasma leakage in Lewis lung cancer.
Indirect immunofluorescence of sections of WT and PKN3 KO primary Lewis lung tumors (30 day) immunostained with an anti-fibrinogen antibody (labeled with a green secondary antibody, left columns) and anti-CD31 antibody (labeled with a red secondary antibodies, middle columns). Images are merged in the right columns. Quantification of the degree of colocalization was performed as described in the Supplementary method section. The Pearson coefficient for colocalization of fibrinogen and CD31 in WT mice was 0.59 +/- 0.11, and for PKN3 KO mice it was 0.56 +/- 0.11 (n=6 for each, P value = 0.45).

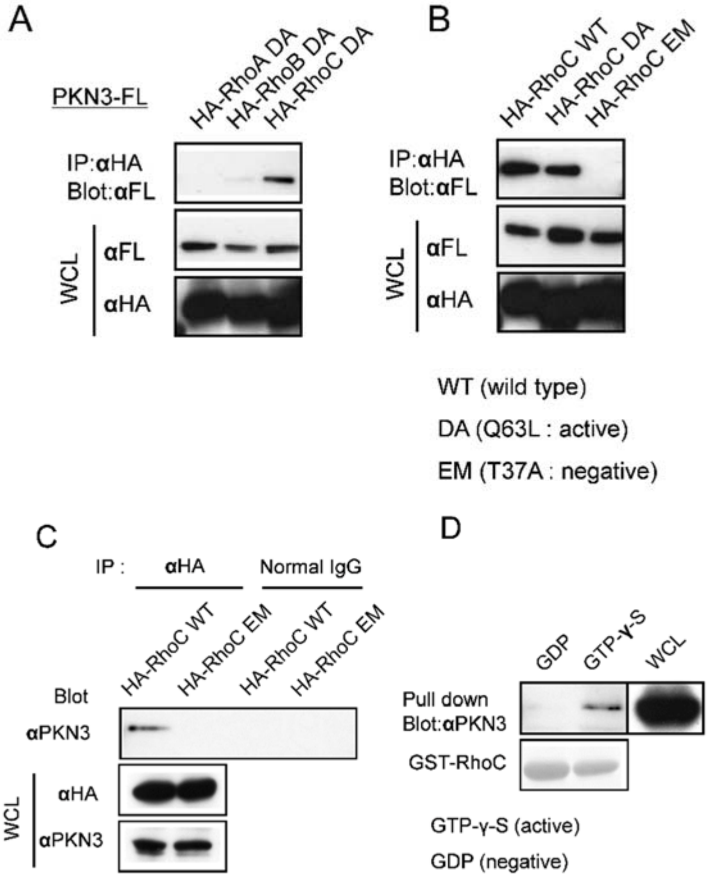

Supplementary Fig. 6. RhoC selectively binds to PKN3 in GTP-dependent manner

"WCL" indicates "whole cell lysate". "DA" and "EM" indicate "dominant active" and "effector-domain mutant", respectively.

(A) Binding of recombinant PKN3 with DA form of RhoC in COS7 cells.

FLAG-tagged PKN3 and HA-tagged DA form of RhoA, RhoB, or RhoC were co-expressed in COS7 cells. Immunoprecipitates with anti HA antibody from soluble lysate of these cells were subjected to immunoblotting with anti FLAG antibody to observe co-precipitated FLAG-tagged PKN3.

(B) Binding of recombinant PKN3 with effector domain mutant form of RhoC in COS7 cells.

FLAG-tagged PKN3 and HA-tagged WT, DA, or EM form of RhoC were co-expressed in COS7 cells. Immunoprecipitates with anti HA antibody from soluble lysate of these cells were subjected to immunoblotting with anti FLAG antibody to observe co-precipitated FLAG-tagged PKN3.

(C) Binding of endogenous PKN3 with RhoC in Hela cells

HA-tagged WT or EM form of RhoC was expressed in Hela cells. Immunoprecipitates with anti HA antibody from soluble lysate of these cells were subjected to immunoblotting with αNUS antibody to detect co-precipitated endogenous PKN3.

(D) Binding of PKN3 with GTPγS or GDP form of RhoC

Purified GST-fused RhoC from bacteria were coupled with GTPγS or GDP, and incubated with Hela cell extract as indicated in the figure. Precipitates with Glutathione Sepharose from the mixtures were subjected to immunoblotting with αNUS antibody to detect co-precipitated endogenous PKN3.

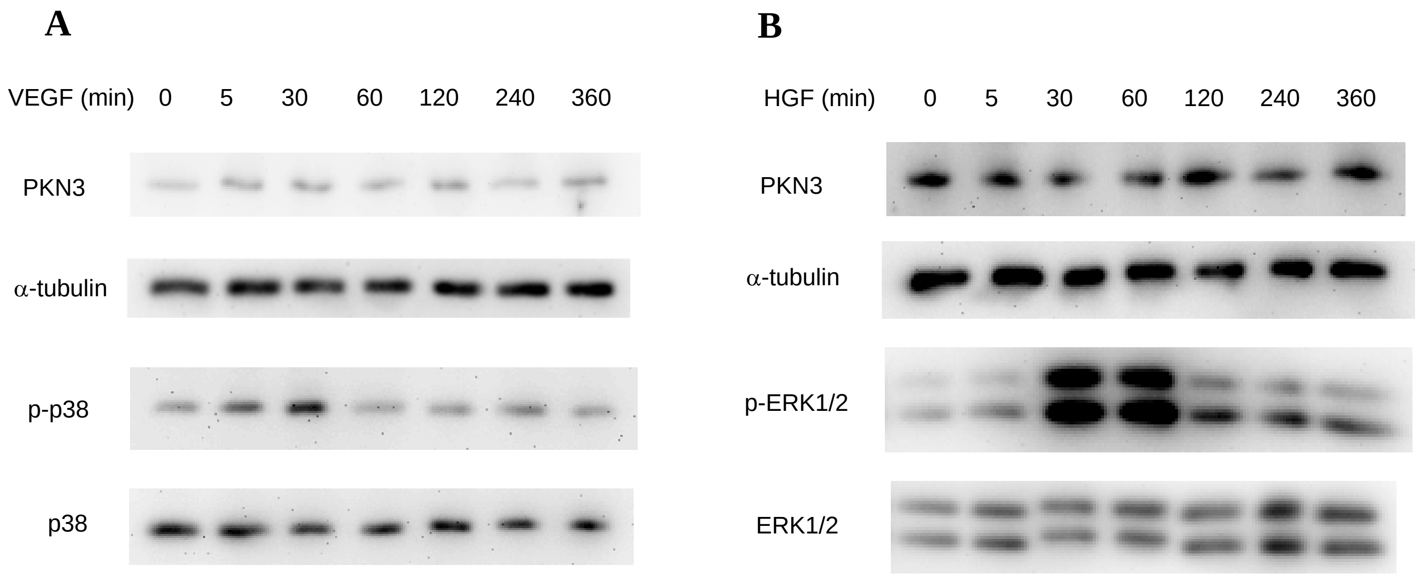

Supplementary Fig. 7. Expression of PKN3 in HUVECs after VEGF or HGF stimulation

Time course of PKN3 expression in HUVECs treated with growth factors. HUVECs (5×10^4) were cultured in complete endothelial cell growth media in 24-well plates. Cells that reached ~80% confluence were starved in EGM2 media containing 0.1% FBS for 16 hours, and then treated with 30 ng/ml of VEGF (A) or 20 ng/ml of HGF (B) for the indicated time. Whole cell lysates were subjected to Western blot analysis for anti PKN3 (αNUS), anti α-tubulin, and phospho-p38 (p-p38), and anti p38, anti phospho-ERK1/2 (p-ERK1/2), and anti ERK1/2 antibodies, respectively. p38 and ERK activations were visualized as hallmarks of the effect of VEGF and HGF on HUVECs. The results shown are representative of three independent experiments. The expression level of PKN3 did not significantly change during HGF or VEGF treatment.

Supplementary Reference

1 Mukai, H. *et al.* Translocation of PKN from the cytosol to the nucleus induced by stresses. *Proc Natl Acad Sci U S A* **93**, 10195-10199 (1996).

2 Schneider, C. A., Rasband, W. S. & Eliceiri, K. W. NIH Image to ImageJ: 25 years of image analysis. *Nat Methods* **9**, 671-675 (2012).

3 Bolte, S. & Cordelieres, F. P. A guided tour into subcellular colocalization analysis in light microscopy. *J Microsc* **224**, 213-232, doi:10.1111/j.1365-2818.2006.01706.x (2006).
